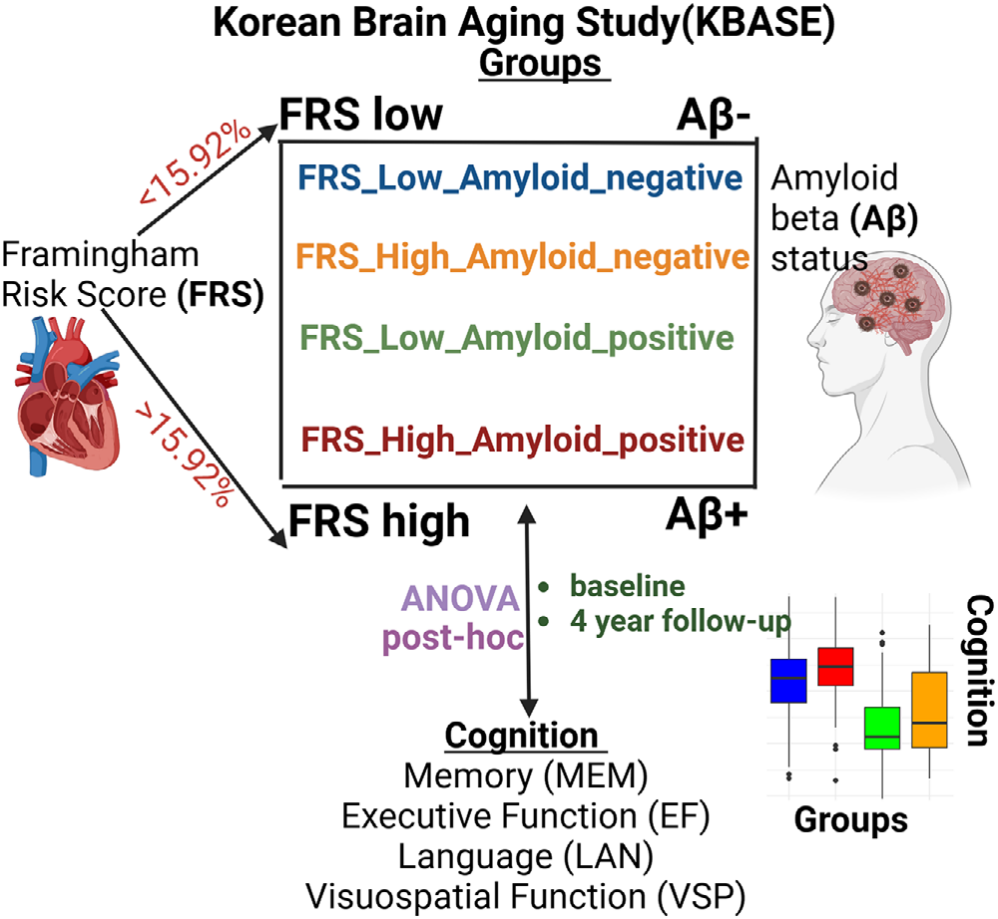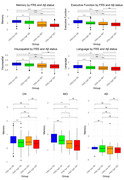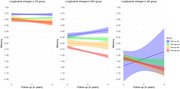# Associations between Amyloid, Cardiovascular Risk, and Cognitive Function in Korean Older Adults: Insights from the KBASE Cohort

**DOI:** 10.1002/alz.094906

**Published:** 2025-01-09

**Authors:** Soumilee Chaudhuri, Desarae A. Dempsey, Yen‐Ning Huang, Sha Cao, Evgeny J. Chumin, Hannah Craft, Paul K. Crane, Shubhabrata Mukherjee, Seo‐Eun Choi, Michael L. Lee, Phoebe Scollard, Jesse Mez, Emily H. Trittschuh, Brandon S Klinedinst, Connie Nakano, Timothy J. Hohman, Dahyun Yi, Min Soo Byun, Shannon L. Risacher, Kwangsik Nho, Andrew J. Saykin, Dong Young Lee, Korean Brain Aging Study for the Early Diagnosis and Prediction of Alzheimer’s Disease (KBASE)

**Affiliations:** ^1^ Indiana Alzheimer’s Disease Research Center, Indianapolis, IN USA; ^2^ Stark Neurosciences Research Institute, Indiana University School of Medicine, Indianapolis, IN USA; ^3^ Indiana Alzheimer's Disease Research Center, Indianapolis, IN USA; ^4^ INDIANA UNIVERSITY, Indianapolis, IN USA; ^5^ Indiana University Network Science Institute, Bloomington, IN USA; ^6^ University of Washington, School of Medicine, Seattle, WA USA; ^7^ Boston University School of Medicine, Boston, MA USA; ^8^ VA Puget Sound Health Care System, Seattle Division, Seattle, WA USA; ^9^ Vanderbilt Memory and Alzheimer’s Center, Institute for Medicine and Public Health, Vanderbilt University Medical Center, Nashville, TN USA; ^10^ Institute of Human Behavioral Medicine, Medical Research Center, Seoul National University, Seoul Korea, Republic of (South); ^11^ Seoul National University College of Medicine, Seoul Korea, Republic of (South); ^12^ Seoul National University Hospital, Seoul Korea, Republic of (South); ^13^ Indiana University School of Informatics and Computing, Indianpolis, IN USA; ^14^ Center for Computational Biology and Bioinformatics, Indiana University School of Medicine, Indianapolis, IN USA; ^15^ Seoul National University Medical Research Center, Seoul Korea, Republic of (South)

## Abstract

**Background:**

Understanding the relationship between cardiovascular burden, amyloid, and cognition in Alzheimer’s disease (AD) is essential for targeted interventions, especially in ethnically diverse populations where research remains limited. This study aimed to investigate these relationships in a cohort of Korean older adults along the AD spectrum.

**Method:**

526 participants from the Korean Brain Aging Study for the Early Diagnosis and Prediction of Alzheimer’s Disease (KBASE) cohort were included in this study. Vascular burden was quantified using Framingham Risk Score (FRS) and participants were categorized into four groups based on combinations of FRS (FRS High or FRS Low with a median split) and amyloid status (Aβ+ or Aβ‐ based on a cut‐off of 1.2373). Cognitive function was evaluated using standardized neuropsychological tests processed with structural equation models to produce domain scores for memory, executive functioning, language, and visuospatial. ANOVA was employed at baseline to analyze cognitive differences among these groups and within each clinical diagnosis. Longitudinal mixed effects models spanning a period of four years from the initial visit captured cognitive changes over time within these groups (Figure 1).

**Result:**

Significant group and pairwise differences were observed among the four groups in all cognitive domains (p < 0.0001). Stratified analysis within each clinical diagnoses group revealed that CN individuals in FRS high Aβ‐ demonstrated significantly lower memory scores compared to those with FRS low Aβ‐ (p < 0.0001), this trend was absent from MCI and AD groups (Figure 2). Longitudinally, FRS high Aβ+ and FRS low Aβ+ groups consistently demonstrated lower memory scores compared to the FRS low Aβ‐ group. Interestingly, no significant difference in cognition was observed between FRS high Aβ‐ and FRS low Aβ‐ groups over time. However, the most pronounced divergence in longitudinal cognition of the four FRS and Amyloid groups was observed within the MCI diagnosis group (Figure 3).

**Conclusion:**

This study highlights the differential impact of cardiovascular risk on cognition depending on amyloid status and clinical diagnosis group. This underscores the importance of considering both cardiovascular risk factors and amyloid pathology early‐on in understanding clinical manifestation and cognitive decline in the AD spectrum, particularly in ethnically diverse populations.